# Effective Gene Trapping Mediated by *Sleeping Beauty* Transposon

**DOI:** 10.1371/journal.pone.0044123

**Published:** 2012-08-31

**Authors:** Guili Song, Qing Li, Yong Long, Qilin Gu, Perry B. Hackett, Zongbin Cui

**Affiliations:** 1 The Key Laboratory of Aquatic Biodiversity and Conservation; Institute of Hydrobiology, Chinese Academy of Sciences, Wuhan, Hubei, People’s Republic of China; 2 Graduate University of Chinese Academy of Sciences, Beijing, People’s Republic of China; 3 Department of Genetics, Cell Biology and Development, University of Minnesota, Minneapolis, Minnesota, United States of America; Wayne State University School of Medicine, United States of America

## Abstract

Gene trapping is a high-throughput approach to elucidate gene functions by disrupting and recapitulating expression of genes in a target genome. A number of transposon-based gene-trapping systems are developed for mutagenesis in cells and model organisms, but there is still much room for the improvement of their efficiency in gene disruption and mutation. Herein, a gene-trapping system mediated by *Sleeping Beauty* (*SB*) transposon was developed by inclusion of three functional cassettes. The mutation cassette can abrogate the splice of trapped genes and terminate their translation. Once an endogenous gene is captured, the finding cassette independently drives the translation of reporter gene in HeLa cells and zebrafish embryos. The efficiency cassette controls the remobilization of integrated traps through inducible expression of *SB* gene. Analysis of transposon-genome junctions indicate that most of trap cassettes are integrated into an intron without an obvious 3′ bias. The transcription of trapped genes was abrogated by alternative splicing of the mutation cassette. In addition, integrated transposons can be induced to excise from their original insertion sites. Furthermore, the Cre/LoxP system was introduced to delete the efficiency cassette for stabilization of gene interruption and bio-safety. Thus, this gene-trap vector is an alternative and effective tool for the capture and disruption of endogenous genes *in vitro* and *in vivo*.

## Introduction

The completion of genome projects for human and other model species has advanced biological researches into the post-genome era. Undoubtedly, the primary task of this era is to elucidate functions of identified genes. Mutagenesis approaches including N-ethyl-N-nitrosourea (ENU)-induced mutations [1], [2], Cre/loxP-mediated gene targeting [3], retrovirus- and transposon-based gene trapping [4], [5], [6], are extensively developed to disrupt expression of genes in model organisms including mouse and zebrafish. ENU treatments can randomly generate point mutations across the target genome and thus lead to mutagenic phenotypes at a high frequency. A number of genes that are essential for the control of various biological processes have been identified by the large-scale ENU mutagenesis screening [7], [8]. Limitations of ENU and other chemical mutagenesis approaches remain the identification of genes whose mutations are responsible for a particular phenotype [9], [10] and the laborious and frustrating tasks of positional cloning. The development of gene targeting is based on homologous recombination and the availability of embryonic stem cells, and this technique is widely used for the generation of knock-out mouse [11], [12]. Since the complete deletion of some genes by conventional gene targeting could be lethal to embryonic development [13], [14], conditional knock-out techinques are alternatively developed [15], [16]; however, the procedures of these approaches are extremely laborious and time consuming.

Gene trapping is an efficient approach for insertional mutagenesis of genes in a target genome. A conventional gene-trap vector consists of a promoterless marker/reporter gene flanked by an upstream splice acceptor (SA) and a downstream poly(A) signal [17], [18], [19], [20]. Insertion of a trapping cassette into an exon or an intron of transcriptional active loci can generate a fusion transcript that contains the upstream exon and the reporter/selectable marker. Since the processed fusion transcript encodes a truncated, often non-functional version of endogenous protein and the marker/reporter, therefore gene trapping is employed to elucidate gene functions by disrupting expression of trapped genes across a target genome, and the integrated trapping cassette serves as a molecular tag for rapid identification and cloning of disrupted gene using the linker-mediated PCR method [21].

Gene trapping and insertional mutagenesis using pseudotyped retrovirus vectors have been extensively employed for elucidation of gene functions in model organisms [22], [23], [24], [25]. These approaches exhibit relative high mutation efficiency, but retroviral vectors demonstrate three disadvantages: their packaging size is limiting, they can induce retroviral-mediated gene silencing and ectopic reporter gene expression [26], [27]. Recently, transposon-based trapping vectors have been developed as alternatives for elucidation of gene functions in mouse and zebrafish [28], [29], [30], [31], [32], [33]. In comparison with viral vectors, transposon-based vectors can carry a large DNA cassette up to 10 Kb [34], [35]. However, overexpressed transposases are harmful to cellular growth and proliferation and can silence the activity of *SB* transposases [34], [36], therefore the transposase used for animal transgenesis is often provided by the translation of *in vitro* synthesized capped mRNA. Thereby, transposons integrated in the genome of transgenic animals are usually less than 10 copies [28], [29]. Analysis of sequencing data indicates that exons make up 1–2% of most vertebrate genome [37] and most transposon-based trap vectors show a great propensity to insert into an intron of target genes [38], [39], [40], so there is less opportunity to directly disrupt endogenous gene expression by a few transposon insertions.

Integration of a trap cassette into an intron is usually expected to interfere with the normal splicing of endogenous transcripts and the mutagenic efficiency mainly depends on the activities of splice acceptor, polyadenylation and transcriptional termination signals in the trapping vector. A weak splice acceptor signal in a trap vector will allow the alternative splicing of endogenous transcript around the trap insertion site and cause the recovery of wild-type transcript, which is one of the major hurdles in creating null mutations using gene traps in mouse [41], [42]. Thus, efficient trapping vectors should be able to truncate the transcription of endogenous genes by the inclusion of a high quality transcriptional termination cassette. Without such a module, splicing around the trap can readily occur and thus result in an insertion without effectively disruption of endogenous gene functions at the insertion locus [29], [43].

The *SB* system is composed of a transposase and a DNA transposon that belongs to the Tc1/mariner superfamily. The *SB* transposase was resurrected through the correction of accumulated mutations in extinct transposase sequences found in the genomes of salmonid fish [44]. Like all other Tc1/mariner transposases, *SB* transposon preferentially inserts into a TA dinucleotides in a recipient DNA sequence and transposes via a “cut-and-paste” mechanism [45]. In addition, *SB* transposase exhibits a high activity and is able to mediate transposition within a wide range of vertebrate cells and tissues [46]. Accordingly, the *SB* transposon system is used for long-term expression in transgenesis [47], [48] and insertional mutagenesis in vertebrates [28], [30], [31], [49]. Moreover, an analysis of 1336 insertion sites in primary and cultured mammalian cells has shown that *SB*-mediated integration exhibits less regional preference than retroviruses and is not significantly influenced by transcriptional activity [38]. Therefore, the *SB* transposon is widely accepted as a powerful tool for insertional mutagenesis and production of transgenic animals.

In this study, we aimed to generate an efficient gene-trapping system using the following strategies: 1) The tilapia *HSP70* promoter was used to drive the expression of SB11 transposase. Inducible expression of SB11 transposase will reduce its cytotoxic effects on cells and model vertebrates as well as allow the remobilization of integrated traps from non-coding sites to new locations and thus increases the opportunity of trapping and mutating endogenous genes [50], [51]. 2) A modified splicing acceptor sequence from the carp (*Cyprinus carpio) β-actin* intron1/exon2 was employed to disrupt the normal splicing of trapped endogenous transcripts. 3) A modified IRES element was introduced to independently drive the translation of reporter gene, which can lead to a six-fold increase in trapping genes [52]. Activities of all components in this system were artificially tested in HeLa cell and zebrafish embryos. It is expected that this novel trapping system would make a great contribution to elucidating functions of many genes that are essential for embryonic development, organogenesis and human diseases in model animals.

## Results

### Generation of a Novel Gene-trap Vector

**Figure 1:**
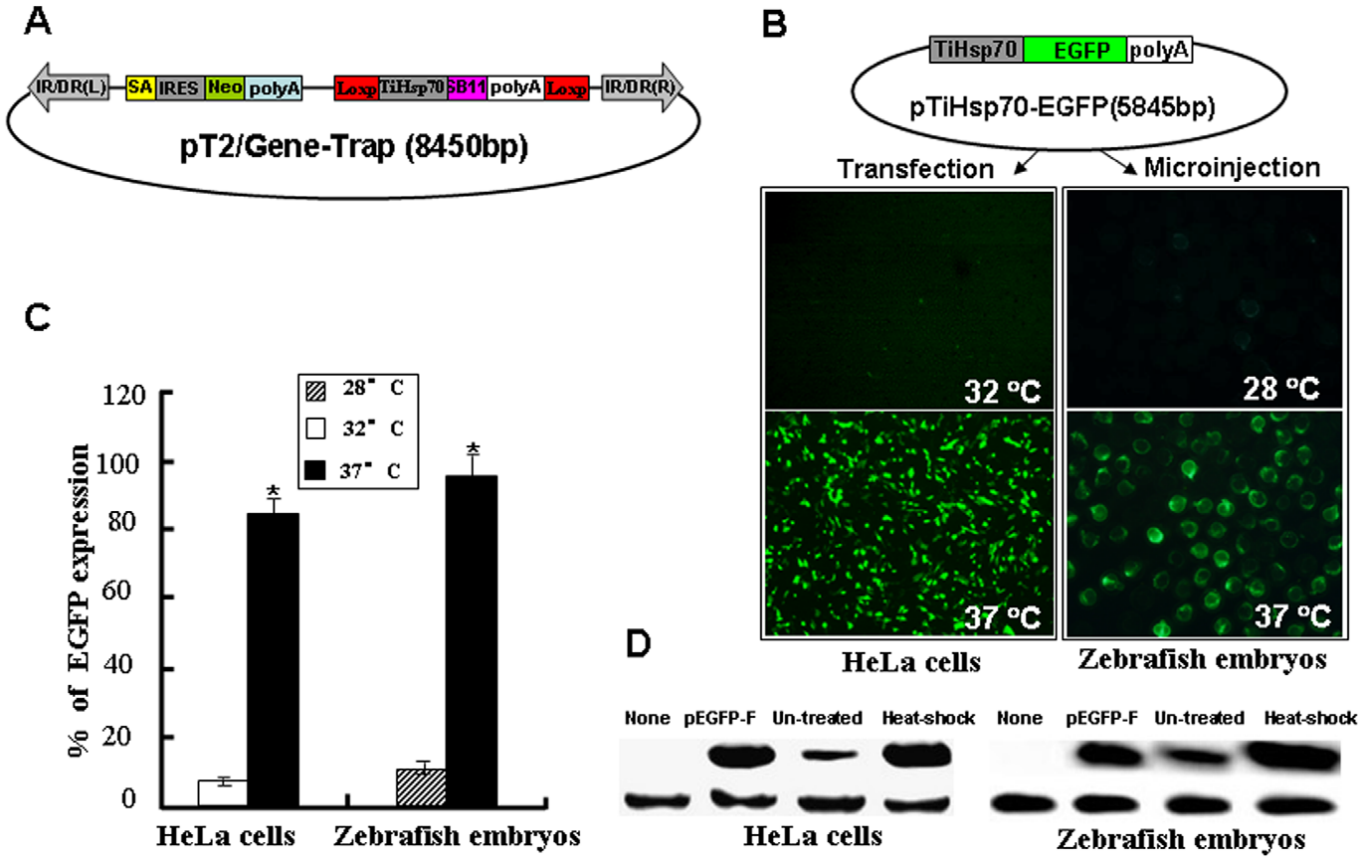
Inducible activity of the tilapia *Hsp70* promoter (*TiHsp70*) at 37 °C**.** (**A**) A novel gene-trap vector mediated by *Sleeping Beauty* transposon. IR/DR(L) and IR/DR(R), left and right inverted repeat/directed repeat of the SB transposon; SA, splice acceptor; IRES, internal ribosome entry site; Neo, kanamycin resistance gene; poly(A), poly(A) signal; TiHsp70, tilapia *Hsp70* promoter; SB11, SB11 transposase gene. (**B**) EGFP was used to monitor the inducible activity of *TiHsp70 in vitro* and *in vivo.* HeLa cells were transfected with pTiHsp70-EGFP at the density of about 80% confluence, treated in medium at 37°C for 1 h and recovered at 32°C for 2 h after transfection. Images were taken under a Nikon TE2000 fluorescent microscope and cell numbers in three fields of view were counted. Zebrafish embryos at one-cell stage were microinjected with pTiHsp70-EGFP. Injected embryos at 24 hpf were incubated in rearing water at 37°C for 1 h and then recovered at 28°C for 2 h. Low magnification fluorescent imaging of zebrafish embryos was performed on a SteReo Lumar V12 microscope form Zeiss and total embryos in three dishes were counted. (**C**) Statistical analysis of EGFP-expressing cells or embryos in (**B**). Data are given as means ± standard Deviation (n = 3). * indicate *P*<0.05 versus the corresponding control. (**D**) Western blot analysis of EGFP in HeLa cells and zebrafish embryos. Heatshock-treated and -untreated cells (transfected with pTiHsp70-EGFP) and embryos (injected with pTiHsp70-EGFP) samples were undergone western blot and the pEGFP-F plasmid was used as a positive control. The expression of reference gene *β-actin* was also analyzed in these samples.

Although there are several versions of transposon-based gene trapping vectors that are used in various vertebrate systems [53], [54], [55], [56], there is room for improvement to increase gene tag and mutation efficiency. Accordingly, we constructed a novel trapping vector pT2/Gene-Trap (Figure 1A) that is mediated by the *SB* transposon system. This vector contains two functional cassettes necessary for improving the efficiency of gene trapping and mutagenesis. In the improved efficiency cassette, expression of SB11 transposase gene was driven by a *Hsp70* promoter from the tilapia genome (*TiHsp70*) [57], which can be activated at 37°C heat treatment. The inducible expression of SB11 conditionally controls the remobilization of integrated trapping cassettes to new target sites in a genome and reduces the cytotoxic effects of *SB* transposase on cells and tissues [50]. In the mutation cassette, a SA signal essential for the proper splice of the carp *β-actin* exon1 and exon2 [58] was inserted upstream of the IRES-Reporter gene such as a neomycin (Neo) or an enhanced green fluorescence protein (EGFP), which is derived from a commercial vector pIRES2-EGFP. Three stop codons required for different reading frames (TGA ATT AGT GA ) were introduced following the SA signal to efficiently truncate the translation of trapped endogenous gene. The utilization of an EMCV/IRES element from Clontech can independently initiate the translation of Neo/EGFP gene once an endogenous gene is trapped and a fusion transcript is formed. This strong splice acceptor signal combined with the polyadenylation signal and transcriptional termination element in our gene-trap vector disrupted the transcription of trapped endogenous genes efficiently.

### Inducible Activity of the Tilapia *HSP70* Promoter

To determine the response of tilapia *HSP70* promoter to a mild heat shock at 37°C, SB11 gene in the improved efficiency cassette was substituted by an EGFP to generate a pTiHsp70-EGFP plasmid (Figure 1B). HeLa cells growing at 32°C were transfected with the pTiHsp70-EGFP. As shown in Figure 1B and 1C, strong fluorescent signals were found in 84% of transfected cells after heat induction for 1 h at 37°C and recovery at 32°C for another 2 h. In contrast, weak signals were seen in about 5% of transfected cells growing at 32°C. To test the *in vivo* activity of tilapia *Hsp70* promoter, zebrafish embryos at one-cell stage were microinjected with pTiHsp70-EGFP and strong EGFP expression was found in 95% of embryos after heat induction for 1 h at 27 hpf, while weak EGFP signals were found in about 10% of non-induced embryos.Western blot analysis was further performed to detect EGFP in both heat shock-treated and un-treated cells (transfected with pTiHsp70-EGFP) and embryos (injected with pTiHsp70-EGFP). As shown in Figure 1D, the expression of EGFP was greatly increased in heat shock-treated cells and embryos, while it was subtle in both un-treated cells and embryos (Figure 1D), indicating that tilapia *Hsp70* promoter is of minimal activity in both cultured cells and zebrafish embryos without heat shock. These data indicate that the tilapia *Hsp70* promoter is sensitive to the mild heat induction at 37°C and is suitable for the conditional control of gene expression *in vitro* and *in vivo*.

### Activity of the Mutation Cassette in an Intron

**Figure 2:**
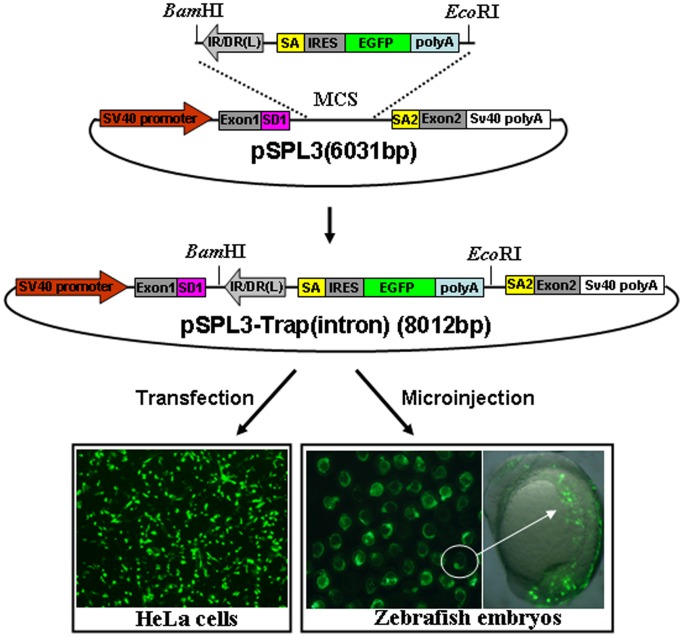
Activity of the trapping cassette in an intron of pSPL3 vector. The trapping cassette was subcloned into the *Bam*HI/*Eco*RI site of pSPL3 vector to generate the pSPL3-Trap(intron), which was used for transient transfection of HeLa cells at 80% confluence. Images were taken under a Nikon TE2000 fluorescent microscope at 48 h after transfection and cell numbers from three independent transfections were counted. Zebrafish embryos at one-cell stage were microinjected with the pSPL3-Trap(intron). Injected embryos at 24 hpf were imaged under a SteReo Lumar V12 microscope form Zeiss and total embryos in three dishes were counted. The ectopic expression of EGFP in one embryo was enlarged and shown in a merged image. SD1, splice donor for exon1; IR/DR(L) and IR/DR(R), left and right inverted repeat/directed repeat of the SB transposon; SA, splice acceptor; IRES, internal ribosome entry site; EGFP, enhanced green fluorescence protein gene; poly(A), poly(A) signal; SA2, splice acceptor for exon2.

The success of gene trapping mainly depends on whether the trap cassette is landed in an endogenous gene or not. The activity of the mutation cassette was examined by subcloning it into the intron of pSPL3 vector, which is originally designed to search genomic DNA for potential exon sequences [59]. As shown in Figure 2, a *Bam*HI*/Eco*RI fragment containing the mutation cassette in Figure 1A was subcloned into the multiple cloning sites (MCS) of pSPL3 to generate a pSPL3-Trap(intron) vector, which was then used for HeLa cells transfection and zebrafish embryo microinjection. Images were taken under a fluorescence microscope after transfection for 24 h and 88% of transfected cells displayed strong EGFP expression. Similarly, strong EGFP expression was found in 92% (n = 1425) of pSPL3-Trap(intron)-injected embryos at 24 hpf.

**Figure 3:**
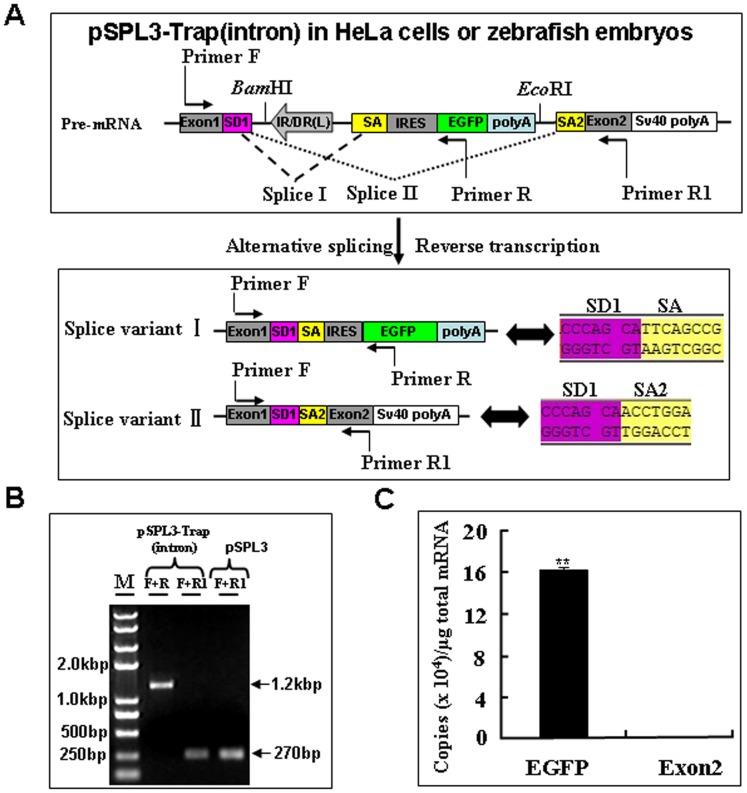
Transcriptional analysis of the trapping cassette in an intron of pSPL3 vector. (**A**) Potential splice variants I and II from the pSPL3-Trap(intron). Sequencing trace files representing the splicing of SD1 with SA and SA1 were shown, respectively. SD1 aSD1, splice donor for exon1; IR/DR(L) and IR/DR(R), left and right inverted repeat/directed repeat of the SB transposon; SA, splice acceptor; IRES, internal ribosome entry site; EGFP, enhanced green fluorescence protein gene; poly(A), poly(A) signal; SA2, splice acceptor for exon2. (**B**) RT-PCR analysis of transcripts from pSPL3-Trap(intron)-transfected HeLa cells. Sequencing results indicate the 1.2-kb band is derived from the splice variant I, which contains exon1, IRES and EGFP, and the 270 bp band results from splice variant II, which represents the proper spicing of exon1 with exon2 in pSPL3. (**C**) The absolute quantification of cDNA using real-time PCR was employed to determine the copy numbers of transcripts including EGFP (F+R, E = 96.1%, R^2^ = 0.9981) and exon2 (F+R1, E = 97.2%, R^2^ = 0.9989). Data are given as means ±standard deviation (n = 3). ** indicate *P*<0.01 versus the exon2 expression level in pSPL3-Trap(intron)-transfected cells.

Since the splice acceptor in gene trap vectors is essential for the formation of fusion transcripts to disrupt the expression of trapped genes, we examined further whether the EGFP expression in HeLa cells and developing embryos (Figure 2) resulted from the proper fusion of EGFP transcript to its upstream exon in the pSPL3. Total mRNA was isolated from HeLa cells transfected with the pSPL3-Trap(intron) vector. Two potential transcript variants I and II, and Sequencing trace files representing the splicing of SD1 with SA and SA1 were shown, respectively (Figure 3A) were analyzed using reverse transcription PCR (RT-PCR). As shown in Figure 3B, a 1.2-kb band was always shown in RT-PCR products of pSPL3-Trap(intron)-transfected HeLa cells and a 270 bp band was occasionally detected by RT-PCR from the same sample. Sequencing results indicate that the 1.2-kb band represents the splice variant I containing exon1, IRES and partial EGFP, and that the 270 bp band is derived from the proper splicing of exon1 with exon2 in pSPL3. Similar results were obtained in zebrafish embryos injected with the pSPL3-Trap(intron) or pSPL3 vector (data not shown).Therefore, the SA signal in our trap vector is able to efficiently direct the proper splicing of reporter gene with an upstream exon in a trapped gene.

To examine further the efficiency of the mutation cassette in disruption of the expression of trapped genes, absolute quantitative real-time PCR assays (qRT-PCR) were performed to determine the copy numbers of transcripts including EGFP and exon2 after reverse transcription of total mRNA from pSPL3-Trap(intron)-transfected HeLa cells [60], [61], [62]. As shown in Figure 3C, the copy numbers of EGFP transcripts is about 1000 times higher than that of exon2 transcript. Similar results were obtained from zebrafish embryos injected with the pSPL3-Trap(intron) vector (data not shown). These data indicate that the mutation cassette in our gene trap vector is able to efficiently disrupt the expression of trapped gene when inserted into an intron.

### Activity of the Mutation Cassette in an Exon

**Figure 4:**
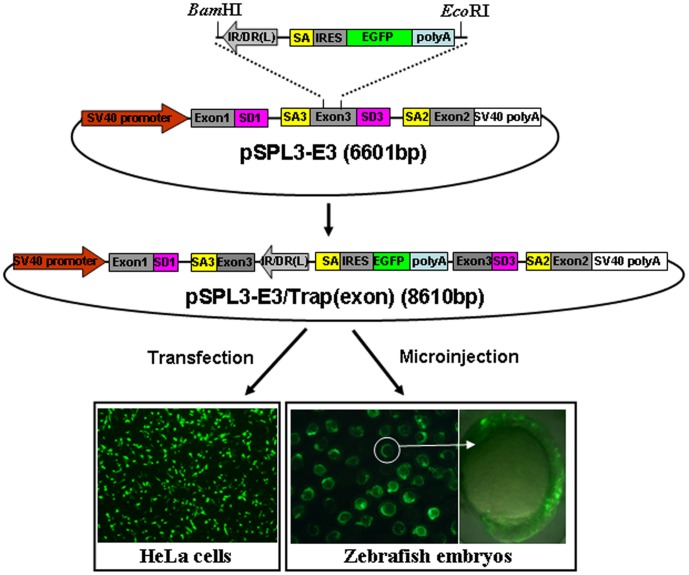
Activity of the trapping cassette in an exon of pSPL3-E3 vector. pSPL3-E3 was generated by insertion of an exon from carp beta-actin gene (Exon3). The trapping cassette was then sucloned into the *Bam*HI/*Eco*RI site of pSPL3-E3 vector to generate the pSPL3-E3/Trap(exon), which was used for transient transfection of HeLa cells at 80% confluence. Images were taken under a Nikon TE2000 fluorescent microscope at 48 h after transfection and cell numbers in three independent transfections were counted. Zebrafish embryos at one-cell stage were microinjected with the pSPL3-E3/Trap(exon). Injected embryos at 24 hpf were imaged under a SteReo Lumar V12 microscope form Zeiss and total embryos in three dishes were counted. The ectopic expression of EGFP in one embryo was enlarged and shown in a merged image. SD1, splice donor for exon1; SA3, splice acceptor for exon3; IR/DR(L) and IR/DR(R), left and right inverted repeat/directed repeat of the SB transposon; SA, splice acceptor; IRES, internal ribosome entry site; EGFP, enhanced green fluorescence protein gene; poly(A), poly(A) signal; SD3, splice donor for exon3; SA2, splice acceptor for exon2.

**Figure 5:**
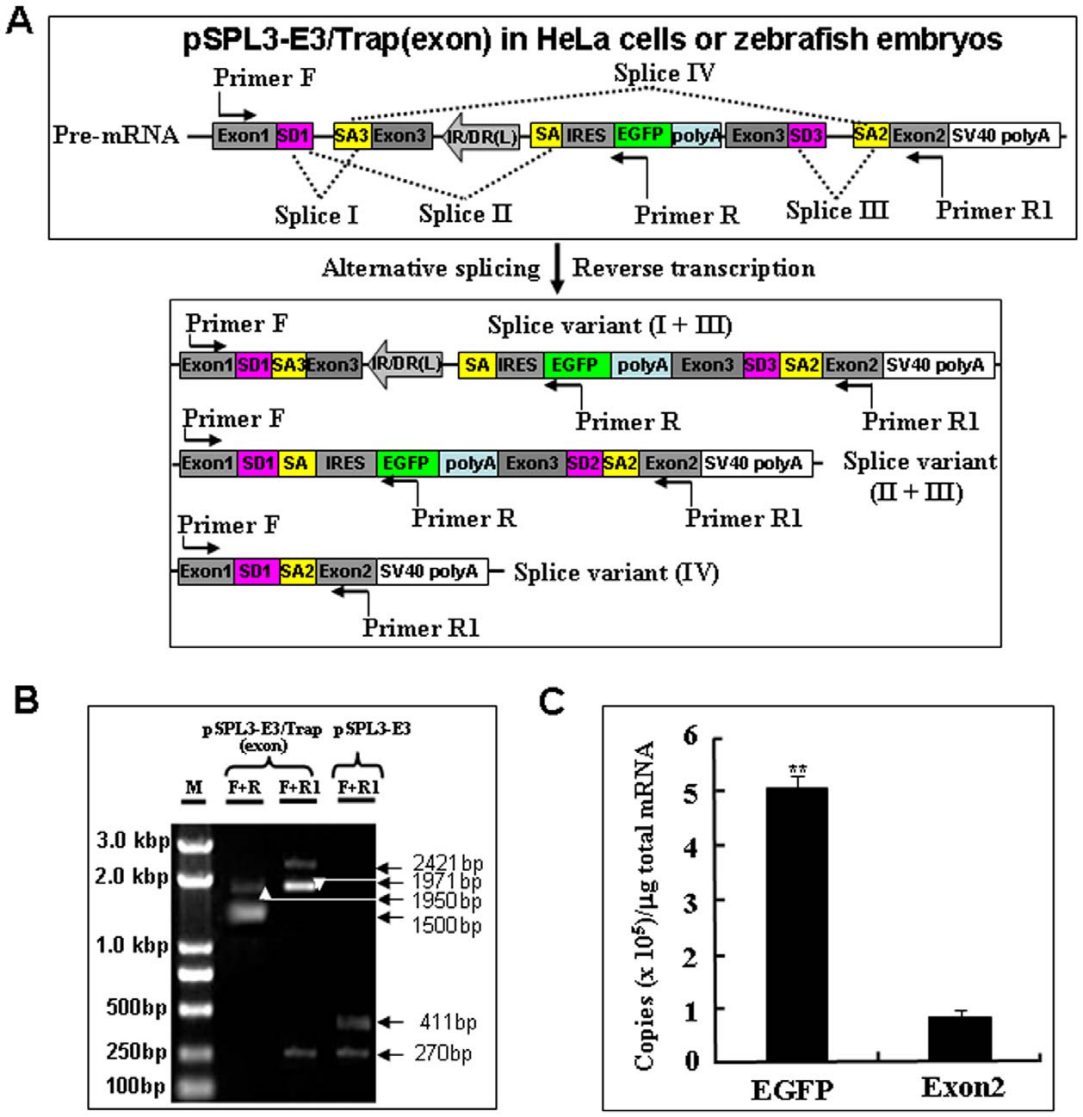
Transcriptional analysis of the trapping cassette in an exon of pSPL3-E3 vector. (**A**) Potential splice variants I+III, II+III and IV from the pSPL3-E3/Trap(exon). SD1, splice donor for exon1; SA3, splice acceptor for exon3; IR/DR(L) and IR/DR(R), left and right inverted repeat/directed repeat of the SB transposon; SA, splice acceptor; IRES, internal ribosome entry site; EGFP, enhanced green fluorescence protein gene; poly(A), poly(A) signal; SD3, splice donor for exon3; SA2, splice acceptor for exon2. (**B**) RT-PCR analysis of transcripts from pSPL3-E3/Trap(exon)-transfected HeLa cells. Sequencing results indicate the 2421 bp and 1950 bp bands are derived from the splice variant I+III, the 1971 bp and 1500 bp bands from the splice variant II+III, and the 270 bp band from splice variant IV. The proper splicing of transcripts from pSPL3-E3 gives rise to a 411 bp band containing exon1, exon3 and exon2, and the 270 bp band containing exon1 and exon2. (**C**) The absolute quantification of cDNA using real-time PCR was employed to determine the copy numbers of transcripts including EGFP (F+R, E = 98.1%, R^2^ = 0.9994) and exon2 (F+R1, E = 97.6%, R^2^ = 0.9976). Data are given as means ±standard deviation (n = 3). ** indicate *P*<0.01 versus the exon2 expression level in pSPL3-E3/Trap(exon)-transfected cells.

To examine the activity of the mutation cassette after its integration into an exon of trapped genes, an exon from the carp *β-actin* gene [58] was subcloned into the MCS of pSPL3 vector to generate a pSPL3-E3 vector (Figure 4). The mutation cassette was introduced at the *Bam*H I/*Eco*R I site in the exon of pSPL3-E3 to obtain the pSPL3-E3/Trap(exon), which was then used for HeLa cell transfection and zebrafish embryo microinjection. Images were taken under a fluorescence microscope after transfection for 24 h and 85% of transfected cells displayed strong EGFP expression. Similarly, strong EGFP expression was seen in about 90% of embryos injected with the pSPL3-E3/Trap(exon) vector. To investigate whether the EGFP expression resulted from the proper activity of the carp β-actin SA signal, total mRNA was isolated from pSPL3-E3/Trap(exon)-transfected HeLa cells and three potential transcript variants (I+III, II+III and IV) in Figure 5A were analyzed using RT-PCR. As shown in Figure 5B, two bands (1950 bp and 1500 bp) were amplified using the primer pair (F+R) and three bands (2421 bp, 1971 bp and 270 bp) using the primer pair (F+R1) from the cDNAs of pSPL3-E3/Trap(exon)-transfected HeLa cells. In addition, two PCR products (411 bp and 270 bp) were obtained from the pSPL3-E3-transfected HeLa cells. Sequencing data indicate that the 2421 bp and 1950 bp bands are derived from the fusion splice variant I +III, the 1971 bp and 1500 bp bands from the fusion transcript II+III, the 411 bp band from the proper splicing of exon1, exon3 and exon2 in pSPL3-E3, and the 270 bp band from the proper splicing of exon1 and exon2 in pSPL3 and pSPL3-E3. Similar results were obtained from zebrafish embryos injected with pSPL3-E3/Trap(exon) and pSPL3-E3 vectors (data not shown). Thus, the SA signal in our trap vector can wield the proper splicing of reporter gene with an upstream splice donor signal (SD) in a trapped endogenous gene. In addition, the insertion of the trap cassette completely abolished the proper expression of trapped exons.

To detect further whether the mutation cassette can disrupt the expression of its downstream exon in trapped gene, absolute qRT-PCR was performed to determine the copy numbers of distinct transcripts in pSPL3-E3/Trap(exon)-transfected HeLa cells [60], [61], [62]. As shown in Figure 5C, the copy numbers of EGFP transcript is about five times higher than that of exon2 transcript, suggesting that insertion of the mutant cassette in an exon markedly inhibited the expression of downstream exons. Similar results were obtained from zebrafish embryos injected with the pSPL3-E3/Trap(exon) vector (data not shown).

Taken together, these data indicate that the mutation cassette in our gene trap vector is able to efficiently knockdown the expression of trapped genes by direct disruption of trapped exon and inhibition of downstream exon splicing.

### An IRES Element is Required for Independent Expression of Reporter Gene

**Figure 6:**
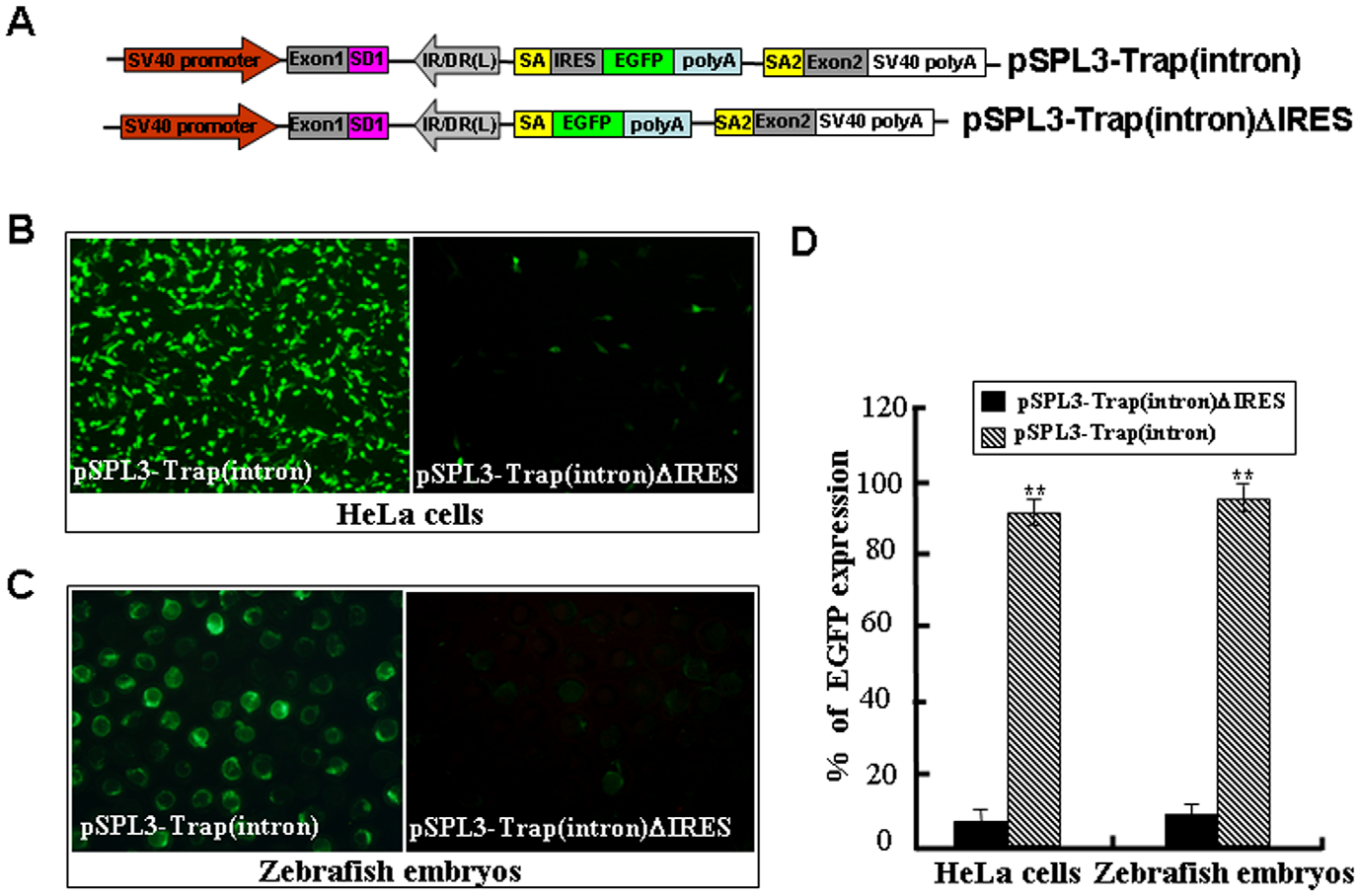
An IRES sequence is required for independent expression of the reporter gene. (**A**) The ECMV/IRES element in the pSPL3-Trap(intron) vector was deleted to generate the pSPL3-Trap(intron)ΔIRES. SD1, splice donor for exon1; IR/DR(L) and IR/DR(R), left and right inverted repeat/directed repeat of the SB transposon; SA, splice acceptor; IRES, internal ribosome entry site; EGFP, enhanced green fluorescence protein gene; poly(A), poly(A) signal; SA2, splice acceptor for exon2. (**B**) pSPL3-Trap(intron) and pSPL3-Trap(intron)ΔIRES constructs were transfected into HeLa cells at 80% confluence, respectively. Images were taken under a Nikon TE2000 fluorescent microscope at 48 h after transfection and cell numbers in three independent transfections were counted. (**C**) Zebrafish embryos at one-cell stage were microinjected with the pSPL3-E3/Trap(exon). Injected embryos at 24 hpf were imaged under a SteReo Lumar V12 microscope form Zeiss and total embryos in three dishes were counted. (**D**) Statistical analysis of EGFP-expressing cells in (**B**) or embryos in (**C**). Each construct was tested three times and each experiment was done in triplicate. Data are given as means ± standard Deviation. ** indicate *P*<0.01 versus the corresponding control.

Although the splice acceptor signal plays an important role in disrupting endogenous gene expression, an IRES element is indispensable to the selective/reporter gene expression. IRES-based vectors are able to capture a wide range of genes expressed in a variety of tissues and embryos at different developmental stages [52]. The ECMV/IRES has been shown to function in developing zebrafish [63]. To determine the efficiency of the ECMV/IRES in driving the expression of reporter gene in our gene trap vector, we deleted the ECMV/IRES element from the pSPL3-Trap(intron) vector to generate the pSPL3-Trap(intron)ΔIRES (Figure 6A). Then, the pSPL3-Trap(intron) or pSPL3-Trap(intron)ΔIRES were used for HeLa cell transfection and zebrafish embryo microinjection. As shown in Figure 6B-D, weak GFP signal was found in less than 10% of HeLa cells or embryos introduced with pSPL3-Trap(intron)ΔIRES, but strong GFP expression was seen in more than 90% of HeLa cells or embryos carrying the pSPL3-Trap(intron). Similarly, deletion of the ECMV/IRES of pSPL3-E3/Trap(exon) in Figure 4 markedly reduced the ratio of EGFP-expressing HeLa cells or embryos. These data demonstrate that the ECMV/IRES element in our trap vector works well for independent expression of reporter gene once a gene is trapped and a fusion transcript formed.

### Analysis of Chromosomal Integration Sites

To examine the gene structure at a locus of trapped chromosomes, a modified splinkerette PCR approach was used to amplify genomic DNA fragments adjacent to integrated trapping cassettes from G418-selected individual cell colonies (Figure S2). Eighteen transposition events were obtained from distinct cell colonies (Table 1). Blasting the human genome in the NCBI and ENSEMBL database with these junction sequences indicated that seventeen of them landed in an intron and one of them integrated in an exon at active genomic loci (Table 1). These data are in consistence with the fact that exons and introns comprise 1.5% and 24% of human genome, respectively [64]. Thus, our gene trap vector appears to insert into endogenous genes and the reporter Neo gene are properly expressed to maintain the survival of HeLa cells in medium containing G418.

**Table 1:** Trapped endogenous genes from G418-resistant HeLa cells.

Junction sequences	Gene name	Accession number	Chromosomename	Exon number	Insertion sites
gtgtctctcctatc**TA** *cagttgaag*	*IPNN5B*	NM_005540.2	Chr:1	18	intron1
ttggattttataca**TA** *cagttgaag*	*APAF1*	NG_029094.1	Chr:12	27	intron16
aaactctttacata**TA** *cagttgaag*	*HERC2*	NM_004667.4	Chr:15	93	intron66
ccctttttgttaac**TA** *cagttgaag*	*MPP2*	NM_005374.3	Chr:17	13	intron3
tctctttcacaaac**TA** *cagttgaag*	*TRIO*	NT_006576.16	Chr:5	57	intron2
tgacagaacataga**TA** *cagttgaag*	*C4orf32*	NM_152400.2	Chr: 4	2	intron1
atacatcttatagt**TA** *cagttgaag*	*ABCB11*	NG_007374.1	Chr: 2	15	intron5
gcacccactgtaca**TA** *cagttgaag*	*CPZ*	NM_001014447.2	Chr:4	11	intron3
tccaacaccacatc**TA** *cagttgaag*	*CACNA1E*	NM_001205293.1	Chr:1	48	intron6
agaatattctgcat**TA** *cagttgaag*	*RP11-553P9.3*	NT_016354.19	Chr:4		5′UTR
gttccttcccaacc**TA** *cagttgaag*	*GRXCR1*	NG_027718.1	Chr:4	4	intron1
tagcattggggagc**TA** *cagttgaag*	*U6snRNA*	NW_001838848.1	Chr: 2	N/A	N/A
tttataatgactta**TA** *cagttgaag*	*TMEM26*	NM_178505.6	Chr:10	7	intron1
tgaggggaaaaata**TA** *cagttgaag*	*TPST2*	NM_001008566.1	Chr:22	7	exon7
tactatcttgttac**TA** *cagttgaag*	*IMMT*	NM_006839.2	Chr:2	15	intron3
tctaagaattcacc**TA** *cagttgaag*	*RPL2I*	NT_024524.14	Chr:13	6	intron1
ttcagctgagtaca**TA** *cagttga*ag	Unknown	AL139023.6	Chr:14	N/A	N/A
taaaatcaatctta**TA** *cagttgaag*	*DENNEB*	NM_144977.4	Chr: 1	20	intron17

Junction sequences were obtained by the splinkerette PCR. Partial trap cassette sequences in italic were shown on the right of TA and partial genome sequences in regular on the left of TA.

**Figure 7:**
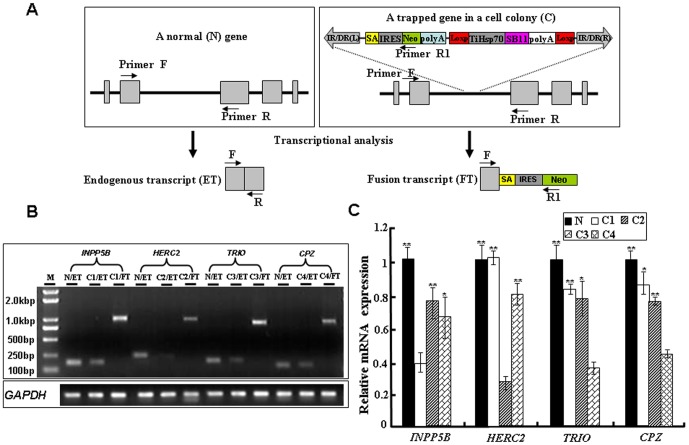
Integration of the SB-based gene trap efficiently disrupts the expression endogenous genes in HeLa cells. (**A**) Schematic representation of a gene trapping insertion in an endogenous gene and potential transcripts in Hela cells. Endogenous exons are boxed and arrows indicate the positions of primers used for transcript analysis. IR/DR(L) and IR/DR(R), left and right inverted repeat/directed repeat of the SB transposon; SA, splice acceptor; IRES, internal ribosome entry site; Neo, kanamycin resistance gene; poly(A), poly(A) signal; TiHSP70, tilapia *Hsp70* promotor; SB11, SB11 transposase gene. (**B**) RT-PCR analysis of transcripts from a trapped endogenous gene (*INPP5B*, *HERC2*, *TRIO* or *CPZ*) in four cell colonies. N: Normal HeLa cells; C: cell colonies; ET: Endogenous transcript; FT: Fusion transcript. The *GAPDH* is used as the control for equal amount of cDNA template in PCR reactions. (**C**) Assessing the mutagenicity of gene-trap insertions by qRT-PCR. Total RNA was isolated from each cell colony and subjected to qRT-PCR analysis. The mRNA expression levels of an endogenous gene in the normal Hela cells and two other colonies without insertion at the gene loci (the controls) were compared to that in a cell colony containing an insertion at the corresponding gene loci. N represent normal HeLa cells; C1 represents *INPP5B* gene; C2 represents *HERC2* gene; C3 represents *TRIO* gene; C4 represents *CPZ* gene. Data are given as Means ± Standard Deviation (n ≥3). ** and * indicate *P*<0.01 and *P*<0.05 versus the controls, respectively.

The success of an insertional mutagenesis approach mainly depends on whether the expression of endogenous genes is efficiently disrupted. Previous studies have shown that insertion of foreign DNA into most locations on a vertebrate genome has little or no effect on any gene or gene product [28], [29]. To examine the effect of our trap vector on disruption of endogenous gene expression at the insertion site, RT-PCR was performed with mRNA samples isolated from four cell colonies growing in medium containing G418 (Figure S2). One trapped gene locus in each cell colony was selected and analyzed using a pair of primers (F+R or F+R1) in Figure 7A. As shown in Figure 7B, a small DNA fragment (100–250 bp) was detected in normal HeLa cells (N/ET) and in each of cell colonies (C1/ET to C4/ET), while a large DNA fragment (∼1-kb) was only obtained from each of colonies (C1/FT to C4/FT). Sequencing results indicate that the small fragments represent the endogenous transcript of *INPP5B*, *HERC2*, *TRIO* or *CPZ* and the large fragments are derived from the fusion transcript of *INPP5B*, *HERC2*, *TRIO* or *CPZ* with the Neo gene. These data indicate that the reporter gene in our gene trap vector is correctly spliced to the trapped endogenous gene in each of these cell colonies and that the trapped gene in each cell colony is heterozygotic at the insertion site.

To further examine whether the expression of four trapped genes was affected by the insertion of a trap cassette, qRT-PCR was conducted by using mRNA samples from normal HeLa cells (N) and cell colonies (C1–C4). The expression levels of these four trapped genes reduced to 30%–45% of those in normal HeLa cells and was significantly lower than those in normal Hela cells and other cell colonies (*P*<0.01 or <0.05 in all cases) (Figure 7C). Therefore, our *SB*-based gene trapping system is suitable for the monitor and interruption of gene expression and functions.

### Remobilization of Gene Trap Cassettes

**Figure 8:**
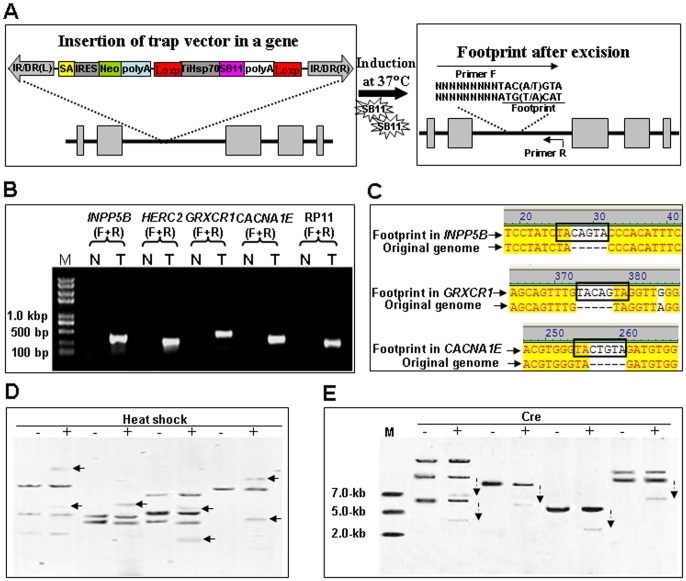
Remobilization of trap inserts in the HeLa cell genome. (**A**) A schematic diagram of trap cassette remobilization and a canonical footprint left in the original insertion site. Arrows indicate the primer containing the footprint (F) and a gene-specific primer (R). IR/DR(L) and IR/DR(R), left and right inverted repeat/directed repeat of the SB transposon; SA, splice acceptor; IRES, internal ribosome entry site; Neo, kanamycin resistance gene; poly(A), poly(A) signal; TiHSP70, tilapia *Hsp70* promoter; SB11, SB11 transposase gene. (**B**) Individual cell colony containing a trap insertion at shown gene loci (*INPP5B*, *HERC2*, *GRXCR1*, *CACNA1E* or *RP11*) was cultured at 32°C in two 35 mm dishes wells. Cells on one dish (T) was subjected to heat induction at 37°C and cells on another dish (N) was kept at 32°C. Total genome DNA was isolated and subjected to PCR analysis using gene-specific primers (F+R) in Table S1. (**C**) Sequencing trace files of independent remobilization events in cell colonies. These sequencing trace files representing independent remobilization event in genes named *INPP5B*, *GRXCR1* and *CACNA1E.* After the excision and remobilization of the gene-trap vector from the original insertion genome locus, footprint was generated as shown in bold box. (**D**) Southern hybridizations. Neo probes were used to detect the copy number of transposons in the genome of HeLa cells incubated at 32°C (-) and the gene-trap cassettes excised from their insertion sites after heat shock (+) at 37°C for 48 h and recovered at 32°C for 5 days. Arrows point to newly-generated inserts after heat shock. (**E**) Southern blotting analysis with Neo probes indicated that the size of genomic DNA fragments with the trap cassette (dashed arrows) reduced by the transient expression of the Cre recombinase (+).

Theoretically, two approaches can be used to improve the efficiency of *SB* transposon-based gene trapping: 1) increase the numbers of gene trap cassettes in a target genome; 2) allow the remobilization of integrated trap cassettes to new genomic sites [51]. Since less than 10 copies of transposons are usually found in the genome of transgenic animals, we tried to improve the gene trapping efficiency by inducting the remobilization of integrated trap cassettes in individual cell colony. Five cell colonies named *INPP5B*, *HERC2*, *GRXCR1*, *CACNA1E* and *RP11* growing at 32°C (N) were induced at 37°C (T) for at least 48 hours to allow the expression of SB11 transposases. Total genomic DNA of cells was isolated and subjected to an excision assay as described previously [65], [66], using a reverse primer (R) against the target genomic DNA and a forward primer (F) containing the typical *SB* footprints TAC(A/T)GTA at its 3′-terminus and the target genome sequence (Figure 8A). The excision of a *SB* transposon from its original integration site gave rise to a PCR product in cells at 37°C, but not in cells at 32°C (Figure 8B). Sequencing results indicate that all of PCR products from the excision sites contain a typical TA-flanked footprint sequence TAC[A/T]GTA (Figure 8C), which is consistent with the activity of *SB*-mediated DNA cleavage [66]. The remobilization of trap inserts was further analyzed with southern blotting. As shown in Figure 8D, DNA bands representing new trap inserts were detected from digested genome of colonies named *INPP5B*, *HERC2*, *GRXCR1*and *CACNA1E* after heat shock at 37°C for 48 h and recovery at 32°C for 5 days, indicating that the integrated gene-trap inserts are excised from the original integration site and new integration patterns have happened after the heat-induced expression of SB11 transposase. These findings suggest that the induced remobilization of trap inserts in a target genome would provide new opportunities for excised trap inserts to reintegrate into other coding regions. The relocation of trap inserts from non-coding regions would significantly improve the efficiency of our gene-trapping vector in animal models.

To avoid the bio-safety concerns and the remobilization of trap inserts that may lead to the normal expression of trapped genes, the Cre recombinase was transiently expressed in G418-selected cell colonies named *INPP5B*, *CACNA1E*, *RP11*and *HERC2* to delete the LoxP-TiHsp70-SB11-poly(A)-LoxP cassette in our gene-trap vector. As shown in Figure 8E, southern hybridization with the Neo probe indicated the proper reduction in size of DNA inserts after transient expression of Cre recombinase. These data suggest that the SB11 cassette can be conditionally deleted to stabilize the phenotypes of mutants.

### The Activities of pT2/Gene-Trap in Transgenic Zebrafish

**Figure 9:**
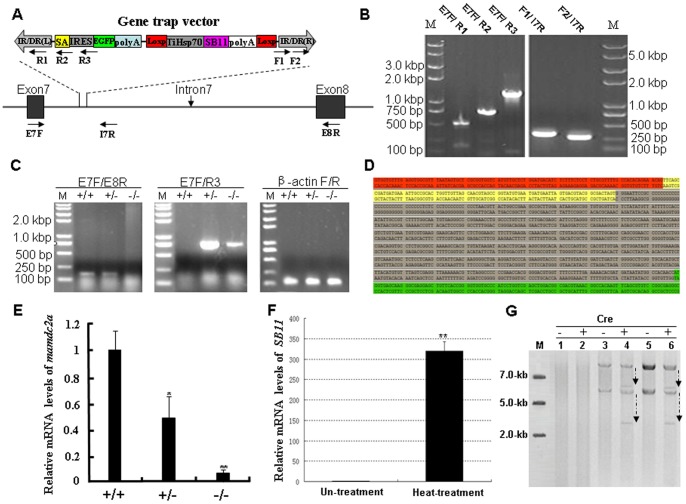
Molecular analysis of the trapped gene *mamdc2a* in transgenic zebrafish. (**A**) Schematic representation of the insertion of gene trap cassette in the intron7 of gene. The locations of primers used for molecular analysis are indicated. (**B**) Integrity analysis of gene trap cassette in transgenic fish. Genomic DNA was extracted from the trail of transgenic fish and subjected to PCR analysis with primes indicated in (**A**). (**C**) RT-PCR analysis of *mamdc2a* transcript in wild-type (+/+), heterozygous (+/−) and homozygous (−/−). Primers E7F/E8R were used to amplify the endogenous transcript and primers E7F/R3 were used to amplify the exon7-EGFP fusion transcript. The *β-actin* transcript was amplified as an internal control. (**D**) The fusion transcript generated from the proper splicing of EGFP gene and its upstream exon in *mamdc2a* gene. Sequences in red represent exon7 of *mamdc2a* gene. Sequences in yellow represent the SA signal. Sequences in gray indicate the IRES followed by the partial EGFP sequence in green. (**E**) qRT-PCR was performed with primers E7F/E8R to determine the expression of endogenous *mamdc2a* gene in wild-type, heterozygous and homozygous embryos at 72 hpf. The *mamdc2a* levels were normalized to the *β-actin* mRNA levels. (**F**) qRT-PCR analysis of SB11 expression in 24 hpf embryos from *mamdc2a*-transgenic line after heat shock at 37°C for 3 h and recovery at 28°C for 2 h. (**G**) Detection of Cre-mediated TiHsp-SB11 excision by southern hybridization. Cre reombinase mRNA was microinjected into wild-type, heterozygous and homozygous embryos. “+” represents the presence of Cre recombinase and “−” represents the absence of Cre recombinase. Line1–2: DNA from wild-type. Line 3–4: DNA from heterozygous. Line 5–6: DNA from homozygous.

A stable transgenic zebrafish line was established and splinkerette PCR was performed to detect the transposable events in F1 genome of this line. As shown in Figure 9A, the *mamdc2a* gene consisting of fourteen exons and thirteen introns was mutated by a canonical transposable element from pT2/Gene-Trap and the Gene-Trap cassette has landed in the intron7 of *mamdc2a* gene. The integrity of the gene-trap cassette at the insertion site was then determined by PCR with different primers (Figure 9B). Next, RT-PCR analysis was conducted with mRNA isolated from wild-type (+/+), heterozygous (+/−) and homozygous (−/−) F2 embryos. The endogenous transcripts of *mamdc2a* gene were detected in wild-type and heterozygous but not in homozygous embryos, while the fusion transcripts of EGFP with exon7 of *mamdc2a* gene were found in heterozygous and homozygous but not in wild-type embryo (Figure 9C). The fusion transcript derived from EGFP and exon7 of gene *mamdc2a* was confirmed by DNA sequencing (Figure 9D). Additionally, qRT-PCR was performed with primers E7F/E8R and mRNA isolated from wild-type, heterozygous and homozygous embryos at 72 hpf. The *mamdc2a* transcriptional levels in heterozygous and homozygous were significantly lower than that in wild-type (Figure 9E).

Apart from the molecular analysis of the trapped gene, the remobilization of gene-trap cassette from the intron7 of *mamdc2a* gene was tested after heat shock for 3 h of F2 embryos from the intercross of *mamdc2a*-transgenic F1 fish. As shown in Figure 9F, the SB11 expression in heat-treated F2 embryos was significantly higher than that in non-treated embryos; however, we didn’t find any novel inserts in heat-treated embryos by southern blot analysis due to the short-term treatment that may not allow the generation of detectable new integration events (data not shown). Moreover, the capped Cre reombinase mRNA was microinjected into wild-type, heterozygous and homozygous embryos to determine whether the Cre/Loxp system in our trap vector can be excised for the stabilization of a disrupted endogenous gene. As shown in Figure 9G, ectopic expression of Cre recombinase in developing F1 and F2 embryos at 72 hpf led to the deletion of TiHsp-SB11 cassette from its original insertion site and the generation of smaller hybridization bands in heterozygous and homozygous embryos.

## Discussion

A gene trap cassette generally consists of an upstream splice acceptor, a promoterless reporter or selectable marker and a downstream polyadenylation signal. When inserted into an intron of expressed genes, the trap cassette is transcribed from the promoter of trapped genes and is spliced to form a fusion transcript containing endogenous gene exon(s) upstream of the insertion site and the reporter [21]. Since the transcription is terminated prematurely at the inserted polyadenylation site, the translation of fusion transcript will give rise to a truncated and nonfunctional version of cellular protein and the reporter. Thus, the success of a gene trap cassette allows disrupting and recapitulating the expression of trapped genes and provides a DNA tag for rapid identification of disrupted endogenous genes. In this study, we have developed a novel gene-trap system that proved to be very effective in disrupting gene expression in HeLa cells and transgenic zebrafish. This gene-trap vector is composed of a mutation cassette as well as a temperature inducible cassette used for improved efficiency, which is mediated by our newly developed *SB* transposon and SB11 transposase [34], [67]. In the mutation cassette, the modified splice acceptor signal from the carp *β-actin* gene [58] can properly direct the splice of a downstream reporter gene with an upstream exon at the insertion site. The IRES element is able to initiate the translation of a downstream reporter gene effectively. The normal splicing and expression of trapped endogenous genes in HeLa cells and developing zebrafish embryos are efficiently disrupted by the combination of all elements in the mutation cassette. In the temperature inducible cassette, the *Hsp70* promoter from tilapia genome [57] is used to drive the expression of SB11 transposase in an inducible manner, which could make the remobilization of integrated trap cassettes for the improvement of trapping efficiency and reduce the overproduction inhibition of the *SB* system. Thus, our *SB*-based gene trap system appears to be suitable for efficient analysis of expression patterns and functions of genes in vertebrates.

Transposon-based gene trap vectors have become indispensable tools for insertional mutagenesis in model vertebrates. However, most of gene trap systems exhibit a relatively low efficiency because of the following reasons. First, the activity of functional elements in most of conventional trap vectors is not carefully examined so it is not sure whether an endogenous gene is disrupted or not even if a gene is trapped. Tissue- or cell-specific expression of reporter gene was detected in the first effort on *SB*-mediated gene trapping in zebrafish, but no mutants were finally obtained [28]. *Tol2*-mediated gene trapping was successfully used to trap endogenous genes; however, the insertion of trapping vector failed to abolish the transcription of trapped genes [29]. A plausible work was performed to generate molecularly null mutations in both larval and adult of zebrafish by artificially testing functional cassettes in a gene breaking vector [49]. Second, less than 10 copies of transposons are usually found in a target genome [28], [29], so the performance of targeting a functional gene by low copy of gene trap cassettes remains quite difficult. The remobilization of integrated transposons can generate new insertions and mutations because transposon reinsertions tend to occur around the original insertion sites. Conditionally gene expression and insertional mutagenesis mediated by *SB* transposon had been performed in mouse with the doxycycline-repressible Tet-Off (tTA) system or Cre/Loxp system [51], [68], in which the transposase was conditionally supplied by another transgenic line that seems to be effective but laborious and less economical. In addition, *Tol2* elements were induced to remobilize from their original insertion sites and thus new mutants were generated in zebrafish [69] and *Xenopus* [70]. In this study, this strategy was successfully employed to induce the remobilization of integrated *SB* tranposons in HeLa cells. Last but not the least, many gene-trap vectors include a SA element in front of a reporter/selective marker, in which the reporter/selective marker can only be expressed correctly in case the trap vector insertion was just in correct frame with the endogenous gene’s open reading frame which means there is only one third chance of the reporter/selective gene expression to represent a successfully gene-trapping event. To circumvent this problem, we introduced the IRES element in front of the reporter/selective marker gene, which have been shown greatly improve the number of detectable gene trap events [71]. Thus, several problems facing most of conventional gene trap vectors are largely solved by our gene trapping system, so this gene trap vector could serve as an alternative tool for the insertional mutagenesis in zebrafish and higher vertebrates.

During the past two decades, various gene trap vectors are developed and successfully used for creating libraries of embryonic stem cell lines that harbor mutations in a single gene and can be used for making mice [72]. Presently, approximately 70% of the protein-coding genes in the mouse genome have been disrupted by gene trap insertions [73]. However, the achievement of saturation mutagenesis in a target genome via current transposon-based gene trapping systems remains difficult due to their insertion site preferences and local hopping. The insertion of most transposons in a target genome is nonrandom because of their characteristic preferences for insertion sites at the primary DNA sequence level. For example, the Harbinger3_DR transposon preferentially inserts into a 15-bp consensus sequence AAACACCWGGTCTTT [74], the *PiggyBac* transposon targets the tetranucleotide sequence TTAA, and all of known Tc1/mariner transposons, including *SB*, Frog Prince, Minos and Hsmar1, prefer to integrate into the TA dinucleotides [46]. By contrast, the *Tol2* element does not appear to have a pronounced insertion preference for any primary DNA sequence [40]. In addition, integration of some transposons exhibits hotspots and cold regions on the target chromosomes. For instances, the *PiggyBac* demonstrates a higher preference for integrations in regions surrounding transcriptional start sites and within long terminal repeat elements [75], and the *Tol2* transposon shows a pronounced preference for integration close to transcriptional start sites [40]. By contrast, Tc1/mariner elements exhibit no or weak preference for transcription units [46]. Additionally, one-fourth of the *SB* trap insertions were found to insert in transcriptional units, a rate that is commensurate with random integration [28]. In this study, we demonstrate that the *SB*-mediated gene trap system seems to randomly integrate into introns and exons of target genes. Moreover, local hopping, a phenomenon of chromosomal transposition in which transposons have a preference for landing into cis-linked sites in the vicinity of the donor locus, limits the chromosomal regions accessible to a transposon jumping out of a given chromosomal site, however it may be useful for saturation mutagenesis [76], which appears to be a shared feature of cut-and-paste transposons. It has been shown that the majority (83%) of *Tol2* reinsertions are mapped on chromosomes other than the transposon donor chromosomes and that 9% of local hopping events mapped less than 300 kb away from the donor loci [69]. The *SB* tranposon seems to have a much larger local transposition interval between 5 and 40 Mb [54], [77]. Therefore, the low transposition activity and diverse insertion site preferences of available transposon systems need to be carefully considered before construction of efficient transposon-based trapping vectors for large-scale mutagenesis.

Another limitation of conventional gene trap systems is how to capture the low-level expressed endogenous genes. To address this problem, a novel vector was recently developed to facilitate the recovery of poorly expressed genes in mouse embryonic stem cells by insertion of an osteopontin enhancer into several conventional gene trap vectors [78]. This strategy would be useful for us to improve our trap system for the genome-wide mutagenesis, and our *SB*-based gene trap system can be further optimized by the combination with Gal4/UAS system or Tet-on system to trap endogenous genes expressed at very low levels. In addition, several poly(A) trap systems have been developed to decipher endogenous genes regardless of their expression patterns and levels [79]. However, most of poly(A) trap systems are able to trigger the nonsense-mediated mRNA decay (NMD) [80] and thus fail to disrupt the expression of endogenous genes at the 5′ end. We are developing another *SB*-based poly(A) trapping system, in which the NMD can be reduced using strategies from other trapping vectors [81]. In conclusion, our *SB*-based gene-trap vector can be used as an alternative tool for large-scale mutagenesis in cells and vertebrates and could proved to be an ideal platform for further development of highly active trapping vectors.

## Materials and Methods

### Ethics Statement

The animal protocol for this research was approved by the Animal Care and Use Committee of Hubei Province in China and by the Institutional Animal Care and Use Committee of Institute of Hydrobiology (Approval ID: Keshuizhuan 0829).

### Plasmids

Our gene-trap vector pT2/Gene-Trap has been designed to efficiently tag and break genes (Fig.1A, GenBank accession number: BankIt1516608 Seq1 JQ692169). These cassettes were sequentially subcloned into the second generation of *SB* transposon pT2/HB. An exon-trapping plasmid pSPL3 was utilized for artificially testing the transcriptional and splicing activities of gene trapping cassettes. The *TiHsp70* promoter was obtained from the tilapia genome using primers 5′-CTT GCT AGC GAG CTC ACC GCG AGC ACT CTG-3′ and 5′- GCA CCG GTC TTG ATT GCT TTG ACT TCG-3′ [57], then inserted at the *Nhe*I/*Age*I site upstream of SB11 transposase gene. The SA sequences from carp *β-actin* gene were amplified using primers 5′- CTT GCT AGC GAT TGC AGC ACG AAA CAGG-3′ and 5′- ATG ACG TCG GTA TAC GTA CGT CACTAA TTC-3′ with the incorporation of stop codes (TGA ATT AGT GA ) for three different read frames in the exon sequence. The IRES element in our trap vector was amplified from the pIRES2-EGFP (Clontech) vector and was used to mediate the reporter translation independently. Sequencing data indicate that all of these components were correctly inserted into the pT2/HB vector.

### Cells and Zebrafish Embryos

HeLa cells (ATCC® CCL-2TM) were cultured under atmospheric condition (95% air and 5% CO_2_) at 32°C in Dulbecco’s modified Eagle medium from GIBCO™-Invitrogen Corporation, containing fetal bovine serum (10%, w/w), penicillin (100 u/mL), streptomycin (100 mg/mL) and amphotericin B (2 µg/mL). The culture medium was replaced 2 to 3 times per week. HeLa cells on 35 mm dish at 80% confluence (about 2×10^5^ cells/dish) were transfected with 2 µg DNA.

AB inbred strain of zebrafish (*Danio rerio*) was reared in a recirculating water system and maintained at standard conditions. Naturally fertilized zebrafish embryos were staged by hour postfertilization (hpf) and embryos at one-cell stage were microinjected with plasmids containing components of our gene trap vector to determine their activities *in vivo*. For microinjection in zebrafish embryos, about 200 pg DNA was microinjected in each embryo.

### Western Blot Assays

Cells were lysted in a buffer containing 1% Nonidet P-40, 0.5% Sodium deoxycholate, 1% SDS, 10 mM Sodium orthovanadate, 2 mM PMSF, 20 µg/mL Leupeptin, 2 µM Pepstatin A, and 20 µg/mL of Aprotinin. Embryo samples were prepared according to our previous protocol [82] and western blot analysis was conducted using primary antibodies against GFP or *β-actin* and then probed with HRP-conjugated secondary antibody.

### Transfection and Selection of G418-resistant Cell Colonies

One day prior to transfection, approximate 3×10^5^ cells in 2 mL of culture medium were seeded on 35 mm culture dishes and cultured overnight. Culture cells at 70%–80% confluence were transfected with the Lipofectamine™ 2000 from Invitrogen. Two days after transfection, the cells were trypsinized and 10% of these cells in selective medium containing 600 µg/mL of G418 were evenly seeded onto 10 cm dishes. The selective medium was replaced twice a week until the formation of cell colonies. Different cell colonies were separately harvested and expanded in medium containing 300 µg/mL of G418 for further analysis.

### Splinkerette PCR Assays

Splinkerette PCR assays as described in previous studies [67], [83] were performed to obtain the flanking chromosomal DNA sequences around the insertion sites of *SB* transposons. Genomic DNAs were extracted from individual G418-resistant HeLa cell colonies. The purified genomic DNA was digested with *Sau*3AI and a linker from the annealing of two complementary oligos (Long stand: 5′-CGA AGA GTA ACC GTT GCT AGG AGA GAC CGT GGC TGA ATG AGA CTG GTG TCG ACA CTA GTGG-3′; Short stand: 5′-GAT CCC ACT AGT GTC GAC ACC AGT CTC TAA TTT TTT TTT TCA AAA AAA-3′) was then ligated to the ends of *Sau*3AI-digested genomic DNA. Primary PCR was performed with primers 5′-CGA AGA GTA ACC GTT GCT AGG AGA GACC-3′ and 5′- TTA AAG GCA CAG TCA ACT TAG TGT ATG TAA ACT TCT G-3′ under the following conditions: 1 cycle at 95°C for 1 min; 10 cycles at 95°C for 10 s and 70°C for 2 min, decrease 0.5°C/cycle; 20 cycles at 95°C for 10 s and 65°C for 2 min; 1 cycle at 70°C for 10 min. The first-round PCR products were diluted for the nest-PCR assays with primers 5′-GTG GCT GAA TGA GAC TGG TGT CGAC-3′ and 5′- TGA AAA ACG AGT TTT AAT GAC TCC AAC TTA AG- 3′ under the following conditions: 1 cycle at 95°C for 2 min; 30 cycles at 95°C for 20 s, 61°C for 30 s and 72°C for 2 min; 1 cycle at 72°C for 10 min. The nested PCR products were separated on the 1.5% agarose gel. Specific DNA bands were purified and cloned into pZero2/TA vector for sequencing. The DNA sequences were BLASTed against the human genome in the ENSEMBL and NCBI database.

### Transcriptional Expression Analysis

Total RNA samples were prepared from transfected cells, embryos or individual cell colonies using the TRIZOL reagent from Invitrogen, and treated with RNase-free DNase at 37°C for 30 min and then at 85°C for 10 min. The first-strand cDNAs were reversely transcribed with oligo-dT primers in the RevertAid™ First Strand cDNA Synthesis Kit from Fermentas according to the manufacture’s instructions. Various fusion transcripts of genes from cells transfected with pSPL3-derived vectors and G418-resistant cell colonies were examined under the conditions: 1 cycle at 95°C for 5 min; 30 cycles at 95°C for 30 s, 60°C for 30 s and 72°C for 3 min; 72°C for 10 min. The PCR products were subjected to 1% agarose gel electrophoresis and sequencing. All specific primers are listed in Table S1.

Real-time PCR analysis was performed to determine the copy numbers of transcripts from the components of pSPL3-derived vectors and the expression level of trapped genes in cell colonies using the SYBR® Green Real-time PCR Master Mix from Toyobo on the Bio-Rad iQ5 2.0 machine. Total RNA samples were digested with the RNase-free DNase (Promega) and then cDNAs were synthesized using oligo-dT primers and random primers and M-MuLV Reverse transcriptase from Fermentas. An absolute quantification method was used to measure the copy numbers of transcripts from pSPL3-derived vectors. A ten-fold dilution series containing 10^2^–10^6^ copies of molecules was prepared from a template sample of known concentration, such as pSPL3-Trap(intron) and pSPL3-E3/Trap(exon) respectively for intron test and exon test, which were shown in Figure S1. The serial ten-fold dilutions and samples were then assayed in the same run. A standard curve was obtained by plotting cycle threshold (Ct) values against log-transformed concentrations of serial ten-fold dilutions (Figure S1). The copy numbers of transcripts in each sample were calculated through a comparison of Ct values from the standard curve.

qRT-PCR was used for expression analysis of trapped genes in cell colonies. Gene-specific primers (22 to 25-mer, Table S1) that span at least one intron were designed to amplify a 150–200 bp fragment from the genomic DNA flanking the *SB* insertion sites. PCR reactions were run in triplicates on 96-well plates and each reaction contains 5 ul of diluted (1∶10) cDNA template from 2 µg of total mRNA, 100 nM of each primer and 10 ul of 2× SYBR Green I Master Mix in a volume of 20 µl. The reaction conditions are as follows: 1 cycle at 95°C for 3 min; 40 cycles at 95°C for 10 s and 60°C for 30 s; 26 cycles at 70°C for 30 s. The expression of *GAPDH* was used as the reference to calculate the relative expression of trapped genes in HeLa cell colonies using the 2^–ΔΔCT^ method [84]. Primers used were listed in Table S1.

### Southern Blot Assays

HeLa cells (3×10^5^) were harvested by centrifugation at 1200 r/min for 5 min. The cell pellets were re-suspended in 300 µl 1× PBS buffer (0.8% NaCl, 0.02% KCl, 0.144% Na_2_HPO4, 0.024% KH_2_PO4) and then lysed at 65°C for 6 h by addition of 300 µl DNA extraction buffer containing 10 mM Tris (PH 8.0), 100 mM EDTA (PH 8.0), 0.5% SDS, and 400 µg/mL proteinase K. The total DNA was purified by using the E.Z.N.A.™ Tissue/DNA Kit from OMEGA/Bio-tek. Total genomic DNA (∼20 µg) was digested with the *Eco*RI at 37°C overnight, separated on a 0.7% agarose gel and transferred onto a positively charged nylon membrane from Roche. The probes were obtained from Neo coding sequences by PCR amplification with primer pairs of NEO-F/NEO-R (Table S1), and labeled with the DIG High Prime DNA Labeling and Detection Starter Kit II from Roche. Hybridization and immunological detection were processed according to the manufacturer’s procedures.

### Remobilization of Integrated Transposons

The remobilization of *SB* transposons integrated in the genome of HeLa cells was performed by placing the G418-resistant HeLa cells at 37°C for 3 to 5 days to induce the expression of SB11 transposase gene in the trap cassette. Since the newly formed transposable events occur in a small portion of cells and G418-resistant cells in the same colony may contain the trap cassettes at different loci, we designed two forward primers containing seven base pair sequences (TACAGTA or TACTGTA) at their 3′ ends to amplify a DNA fragment around the footprint of *SB* transposon remobilization using a PCR-based method described previously [66]. PCR assays were performed under the conditions: 95°C for 5 min; 34 cycles at 95°C for 30 s, 58°C for 30 s and 72°C for 30 s; 72°C for 10 min. The PCR products were purified and sequence, primers used in this experiment are listed in Table S1.

### Statistical Analysis

Data were expressed as means ± standard deviation and student’s *t* test was performed using the SPSS version 15.0 for windows (Inc., Chicago, Illinois, USA) to determine the significant difference (*P*<0.05 or *P*<0.01) between two groups.

## Supporting Information

Figure S1 **Standard curves for absolute quantification of EGFP and exon2 transcripts from pSPL3-derived vectors.** A ten-fold dilution series containing 10^2^–10^6^ copies of molecules was prepared from a template sample of known concentration, pSPL3-Trap(intron) and pSPL3-E3/Trap(exon) respectively for intron test and exon test. A standard curve was obtained by plotting cycle threshold (Ct) values against log-transformed concentrations of serial ten-fold dilutions. In pSPL3-Trap(intron)-trap test, the primer amplification efficiencies for EGFP and exon2 are 96.1%, and 97.2%, R^2^ are 0.9981 and 0.9989, respectively(**A,B**). In pSPL3-E3/Trap(exon)-trap test, the primer amplification efficiencies for EGFP and exon2 are 98.1% and 97.6%, R^2^ are 0.9994 and 0.9976, respectively(**C,D**). Copy numbers of transcripts in samples were calculated through a comparison of Ct values from the standard curve.
(TIF)

Figure S2 **Schematic overview of the experimental procedure for gene trap analysis in HeLa cells.** Gene trap vector pT2/Gene-Trap was transfected into HeLa cells and induced in medium at 37°C for 24 h before G418 selection. After being selected in medium containing 600 µg/mLG418 for three to four weeks, individual cell colonies were separated and expanded for integration site analysis. IR/DR(L) and IR/DR(R), left and right inverted repeat/directed repeat of the SB transposon; SA, splice acceptor; IRES, internal ribosome entry site; Neo, kanamycin resistance gene; poly(A), poly(A) signal; TiHSP70, tilapia *Hsp70* promotor; SB11, SB11 transposase gene.
(TIF)

Table S1 **Primers used in this study.**
(DOC)
