# Supplementary figures and images for: Effective Gene Trapping Mediated by Sleeping Beauty Transposon
Source: PLoS One. 2012 Aug 31;7(8):e44123. doi: 10.1371/journal.pone.0044123 (PMC3432063; doi:10.1371/journal.pone.0044123)

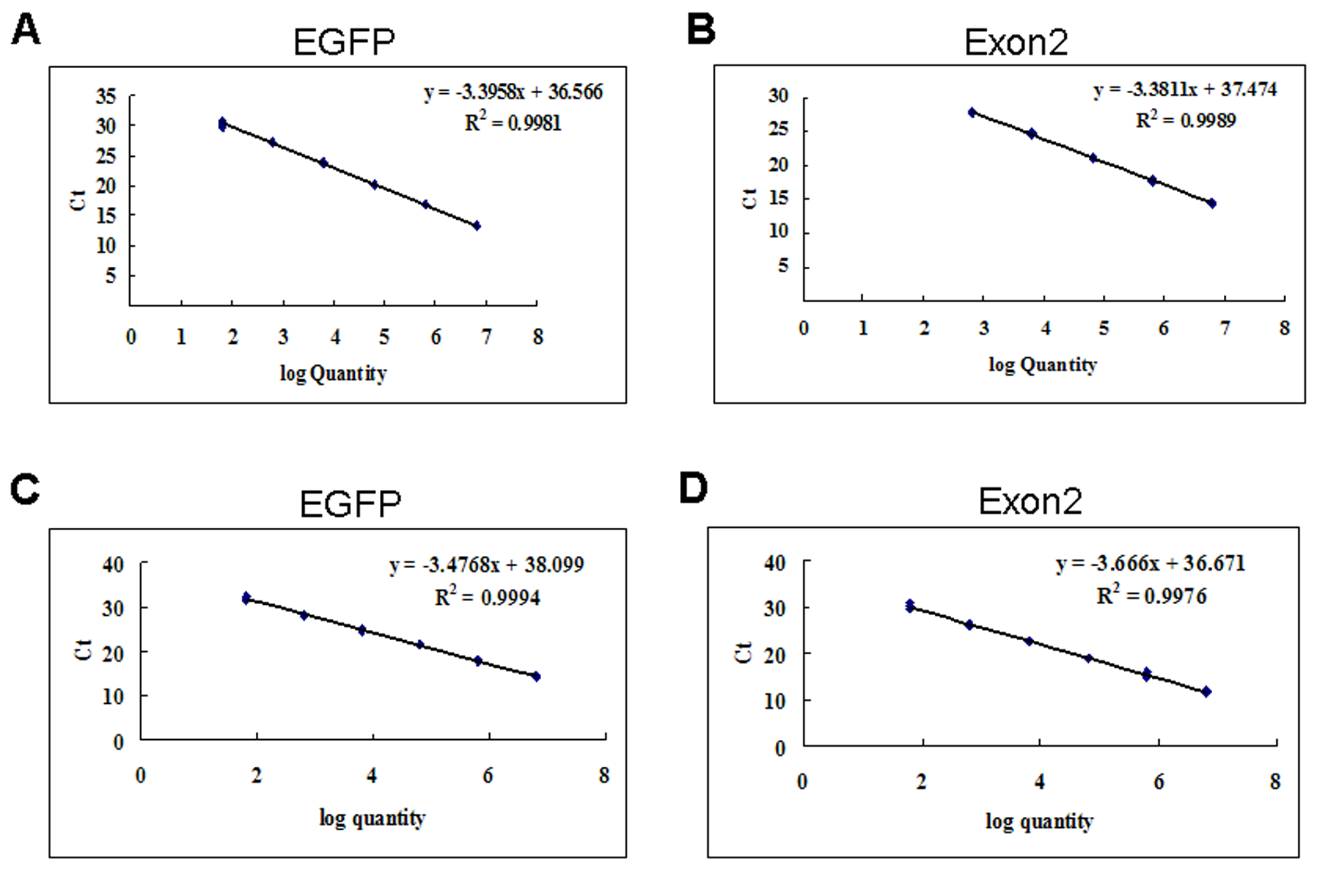

Supplement: Figure S1 — Standard curves for absolute quantification of EGFP and exon2 transcripts from pSPL3-derived vectors. A ten-fold dilution series containing 102–106 copies of molecules was prepared from a template sample of known concentration, pSPL3-Trap(intron) and pSPL3-E3/Trap(exon) respectively for intron test and exon test. A standard curve was obtained by plotting cycle threshold (Ct) values against log-transformed concentrations of serial ten-fold dilutions. In pSPL3-Trap(intron)-trap test, the primer amplification efficiencies for EGFP and exon2 are 96.1%, and 97.2%, R2 are 0.9981 and 0.9989, respectively(A,B). In pSPL3-E3/Trap(exon)-trap test, the primer amplification efficiencies for EGFP and exon2 are 98.1% and 97.6%, R2 are 0.9994 and 0.9976, respectively(C,D). Copy numbers of transcripts in samples were calculated through a comparison of Ct values from the standard curve. (TIF) [file pone.0044123.s001.tif]

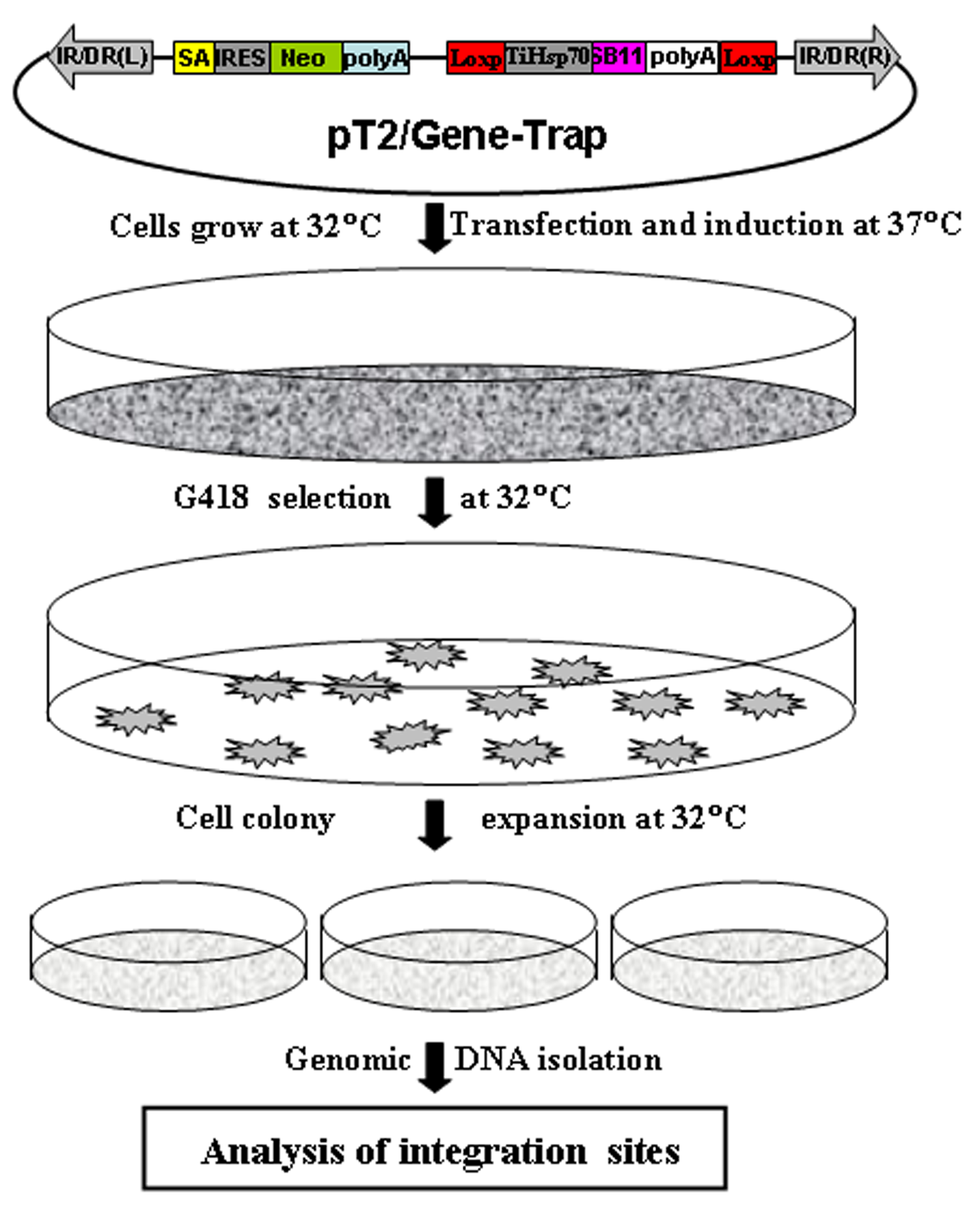

Supplement: Figure S2 — Schematic overview of the experimental procedure for gene trap analysis in HeLa cells. Gene trap vector pT2/Gene-Trap was transfected into HeLa cells and induced in medium at 37°C for 24 h before G418 selection. After being selected in medium containing 600 µg/mLG418 for three to four weeks, individual cell colonies were separated and expanded for integration site analysis. IR/DR(L) and IR/DR(R), left and right inverted repeat/directed repeat of the SB transposon; SA, splice acceptor; IRES, internal ribosome entry site; Neo, kanamycin resistance gene; poly(A), poly(A) signal; TiHSP70, tilapia Hsp70 promotor; SB11, SB11 transposase gene. (TIF) [file pone.0044123.s002.tif]
