# Supplementary material for: Effective Gene Trapping Mediated by Sleeping Beauty Transposon
Source: PLoS One. 2012 Aug 31;7(8):e44123. doi: 10.1371/journal.pone.0044123 (PMC3432063; doi:10.1371/journal.pone.0044123)
Supplement: Table S1 — Primers used in this study. (DOC) [file pone.0044123.s003.doc]

Table S1. Primers used in this study

| **Primer names** | **Sequence (5’-3’)** | **Usage** | **Used in** |
| --- | --- | --- | --- |
| Test-F | tctgagtcacctggacaacctcaaag | Primer F, Transcript analysis | Figure 3A,B and Figure 5A,B |
| EGFP-R | ttacttgtacagctcgtccatg | Primer R, Transcript analysis | Figure 3A,B and Figure 5A,B |
| Test-R | atctcagtggtatttgtgagccagg | Primer R1,Tanscript analysis and | Figure 3A,B and Figure 5A,B |
| Exon2F | cgacaggcccgaaggaat | Quantification PCR | Figure 3C and Figure 5C |
| Exon2R | cctgcacctgaggagtgaatt | Quantification PCR | Figure 3C and Figure 5C |
| EGFPf | gcagaagaacggcatcaagg | Quantification PCR | Figure 3C and Figure 5C |
| EGFPr | cggactgggtgctcaggtag | Quantification PCR | Figure 3C and Figure 5C |
| GE1TF | gcggcgggatgaggag | *INPP5B*-F, Transcript analysis | Figure 7A,B |
| GE1TR | cttggttgcaagctttctgg | *INPP5B*-R, Transcript analysis | Figure 7A,B |
| GE3TF | gccttcagatgctgattcttctgc | *HERC2*-F, Transcript analysis | Figure 7A,B |
| GE3GR | gaggagaacgaggggcactcc | *HERC2*-R, Transcript analysis | Figure 7A,B |
| GE5TF | gtttcgaaaaaacgatgaaatgaaag | *TRIO*-F, Transcript analysis | Figure 7A,B |
| GE5TR | ctgggaatacaggctagataggaaa | *TRIO*-R, Transcript analysis | Figure 7A,B |
| GE8TF | tgccaccgctacttcacgagag | *CPZ*-F, Transcript analysis | Figure 7A,B |
| GE8TR | tcagctcgtgctggccgg | *CPZ*-R, Transcript analysis | Figure 7A,B |
| NeoTR | gcggcatcagagcagccg | Neo-R1, Transcript analysis | Figure 7A,B |
| GE1RT-F | atgtctgccgccgccgg | *IPNN5B*-RT-F, Quantification PCR | Figure 7C |
| GE1RT-R | cttggttgcaagctttctggt | *IPNN5B*-RT-F, Quantification PCR | Figure 7C |
| GE3RT-F | gccttcagatgctgattcttctgc | *HERC2*-RT-F, Quantification PCR | Figure 7C |
| GE3RT-R | gaggagaacgaggggcactcc | *HERC2*-RT-R, Quantification PCR | Figure 7C |
| GE5RT-F | gaaaaaacgatgaaatgaaag | *TRIO*-RT-F, Quantification PCR | Figure 7C |
| GE5RT-R | gaatacaggctagataggaa | *TRIO*-RT-R, Quantification PCR | Figure 7C |
| GE8RT-F | ctgctatgacccgctgggaga | *CPZ*-RT-F, Quantification PCR | Figure 7C |
| GE8RT-R | gcgtaggagtggtggctgaa | *CPZ-RT-R,* Quantification PCR | Figure 7C |
| GAPDH-F | acgcatttggtcgtattggg | Tanscript analysis and Quantification PCR | Figure 7B,7C |
| GAPDH-R | tgattttggagggatctcgc | Tanscript analysis and Quantification PCR | Figure 7B,7C |
| NEO-F | tgtcactgaagcgggaagg | Southern hybridization | Figure 8C |
| NEO-F | cggcgataccgtaaagcac | Southern hybridization | Figure 8C |
| Ge1GF2 | gcagtgcctctcctatctacagta | *IPNN5B* PrimerF, Excision PCR | Figure 8 |
| Ge1GR | aggctgaggtcaggcagtgcag | *IPNN5B* PrimerR , Excision PCR | Figure 8 |
| Ge3GF2 | ccaaaactcttctacatacagta | *HERC2* PrimerF , Excision PCR | Figure 8 |
| Ge3GR | gaggagaacgaggggcactcc | *HERC2* PrimerR , Excision PCR | Figure 8 |
| Ge5GF2 | atttctctttttcacaaactacagta | *TRIO* PrimerF , Excision PCR | Figure 8 |
| Ge5GR | tgtggcccaggatgaccacag | *TRIO* PrimerR , Excision PCR | Figure 8 |
| Ge8GF2 | gagcacccactgtacatacagta | *CPZ* PrimerF ,  Excision PCR | Figure 8 |
| Ge8GR | agggtccacccttctcagattgg | *CPZ* PrimerR ,  Excision PCR | Figure 8 |
| Ge10GF | tgtcagaatattctacattacagta | *RP11* PrimerF , Excision PCR | Figure 8 |
| Ge10GR | cataggagtattttggaggaggaaacatg | *RP11* PrimerR , Excision PCR | Figure 8 |
| F1 | tgacctaagacagggaatttttactaggattaaatg | Integrity analysis of gene trap vector | Figure 9 |
| F2 | tgagtttaaatgtatttggctaaggtgtatg | Integrity analysis of gene trap vector | Figure 9 |
| R1 | ttaaaggcacagtcaacttagtgtatgtaaacttctg | Integrity analysis of gene trap vector | Figure 9 |
| R2 | atgacgtcggtatacgtacgtcactaattc | Integrity analysis of gene trap vector | Figure 9 |
| R3 | tagccatggttgtggccatattatcatcg | Integrity analysis of gene trap vector | Figure 9 |
| E7F | gtggtgtttgaggtggcgtttaatag | Tanscript analysis and Quantification PCR | Figure 9 |
| I7R | tgaaacacttaatgacatctccgtac | Integrity analysis of gene trap vector | Figure 9 |
| E8R | ggtgtgatctccaatgcggtaaac | Tanscript analysis and Quantification PCR | Figure 9 |
| SB11F2 | cactgctccaaaaccgacataag | Quantification PCR | Figure 9 |
| SB11R2 | tcccccttcttcctccaaac | Quantification PCR | Figure 9 |
| β-actinF | tcaccaccacagccgaaag | Tanscript analysis and Quantification PCR | Figure 9 |
| β-actinR | ggtcagcaatgccagggta | Tanscript analysis and Quantification PCR | Figure 9 |
